# Open pancreaticoduodenectomy: setting the benchmark of time to functional recovery

**DOI:** 10.1007/s00423-021-02333-3

**Published:** 2021-09-23

**Authors:** Giovanni Marchegiani, Giampaolo Perri, Stefano Andrianello, Gaia Masini, Giacomo Brentegani, Alessandro Esposito, Claudio Bassi, Roberto Salvia

**Affiliations:** Department of Surgery and Oncology, General and Pancreatic Surgery – The Pancreas Institute, University of Verona Hospital Trust - Verona University, P.le Scuro 10, 37134 Verona, Italy

**Keywords:** Midline incision, Open, Pancreatoduodenectomy, Minimally invasive, Benchmark, Functional recovery

## Abstract

**Purpose:**

No accepted benchmarks for open pancreaticoduodenectomy (PD) exist. The study assessed the time to functional recovery after open PD and how this could be affected by the magnitude of midline incision (MI).

**Materials and methods:**

Prospective snapshot study during 1 year. Time to functional recovery (TtFR) was assessed for the entire cohort. Further analyses were conducted after excluding patients developing a Clavien-Dindo ≥ 2 morbidity and after stratifying for the relative length of MI.

**Results:**

The overall median TtFR was 7 days (*n* = 249), 6 days for uncomplicated patients (*n* = 124). A short MI (SMI, < 60% of xipho-pubic distance, *n* = 62) was compared to a long MI (LMI, *n* = 62) in uncomplicated patients. The choice of a SMI was not affected by technical issues and provided a significantly shorter TtFR (5 vs 6 days, *p* = 0.002) especially for pain control (4 vs. 5 days, *p* = 0.048) and oral food intake (5 vs. 6 days, *p* = 0.001).

**Conclusion:**

Functional recovery after open PD with MI is achieved within 1 week from surgery in half of the patients. This should be the appropriate benchmark for comparison with minimally invasive PD. Moreover, PD with a SMI is feasible, safe, and associated with a faster recovery.

**Supplementary Information:**

The online version contains supplementary material available at 10.1007/s00423-021-02333-3.

## Introduction

Pancreaticoduodenectomy (PD) is among the most complex surgical procedures in the field of gastrointestinal surgical oncology. PD requires technically demanding resection and reconstruction phases that exhibit extreme variability, ranging from straightforward procedures to complex multivisceral resections that include venous and arterial resection [1, 2]. For these reasons, PD has always been managed with a traditional open approach. Midline or transverse subcostal incisions are the most commonly used ways to access the abdominal cavity in cases of PD, but the choice is often based on individual preferences and traditional motivations rather than clinically relevant criteria [3].

Recently, minimally invasive surgery has sensibly expanded in the field of pancreatic surgery. The minimally invasive approach for PD was described for the first time by Gagner and Pomp in 1994 [4]; despite initial unfavorable outcomes in terms of morbidity and mortality, there was a rapid spread of this technique among centers performing advanced minimally invasive surgery and those with a large caseload of hepato-pancreato-biliary procedures. A further boost for minimally invasive PD has come from the introduction of robotic surgery. In the last 10 years, there has been flourishing research activity that has led to evidence that minimally invasive PD is at least associated with reduced blood loss, reduced hospitalization, and, therefore, a more rapid postoperative recovery than the classic open approach [5, 6]. Because the high postoperative management cost, rather than the cost of surgery, seems to be the main factor responsible for the high expense associated with elective PD [7, 8], the high costs of minimally invasive PD could soon be justified by the simultaneous reduction of expenses associated with postoperative hospitalization.

However, in the comparison between open and minimally invasive PD, the focus has always been on a standardized surgical technique for the minimally invasive approach without defining a benchmark for the open counterpart. This represents a significant issue that has been underestimated, assuming that all the types of open access are similar. Several factors may be associated with functional recovery after open PD, such as surgeon experience, center caseload, the presence of standardized clinical pathways, and patient-related and procedure-related factors. Indeed, not all abdominal incisions are the same, and their invasiveness may have specific effects on abdominal wall function, respiratory function, pain, and, subsequently, postoperative recovery [9–11].

The aims of the present study are to explore functional recovery after PD with a classic midline open approach in the setting of a high-volume center with highly standardized perioperative clinical pathways, to establish a possible benchmark for future comparison with minimally invasive PD, and to assess whether the invasiveness of open access could affect functional recovery.

## Materials and methods

### Study population

The present study is consistent with the STROBE (STrengthening the Reporting of Observational studies in Epidemiology) recommendations and was approved by the institutional review board (Comitato Etico delle province di Verona e Rovigo, approval number 1101CESC). We performed a prospective snapshot observational study that included all consecutive elective PDs performed via an open approach during a one-year period (August 2018–August 2019) at the Department of General and Pancreatic Surgery—The Pancreas Institute, University of Verona Hospital Trust, Italy. Demographic, clinicopathologic, and perioperative data were collected.

### Surgical technique

All procedures were standardized and performed by experienced pancreatic surgeons via an MI starting from the xiphoid process up to the supraumbilical region. The skin was incised with a conventional scalpel, and the abdominal wall was dissected by electrocoagulation until the peritoneal cavity was reached. If the MI was elongated down to the umbilical region, the skin was incised in a semicircular direction at the level of the umbilicus. The extension of the MI below the umbilical region was decided at the surgeon’s discretion, to achieve optimal exposure according to patient characteristics (i.e., short xipho-umbilical distance, long distance from body surface to retroperitoneal region). Abdominal closure was also standardized with only the rectus abdominis fascia being closed using interrupted braided absorbable sutures (Vycril, Ethicon, Inc., NJ) with a stitch interval of 1 cm. The skin was directly closed with staples. Subcutaneous sutures were not used. The wound was always covered with a standard sterile dressing. At the end of the procedure, the following measurements were recorded: the xipho-umbilical distance, xipho-pubic distance, and MI length. All measures were reported in centimeters. Postoperative clinical pathways were also highly standardized as already described by our group [12]. Briefly, surgical drains were managed according to selective drain placement and early drain removal protocols. There was no routine intensive care unit admission. Nasogastric tube removal was performed at the end of surgery. Postoperative pain management was achieved through an acute pain service team. Early mobilization occurred the day after surgery. A clear liquid diet was started on postoperative day (POD) 2, and the bladder catheter was removed.

### Objectives and outcomes

The main objective of this study was to assess functional recovery after open PD. The analysis was performed on the entire cohort and then only in patients with an uneventful postoperative course or who only developed mild complications managed in the outpatient setting (Clavien-Dindo < 2; *uncomplicated patients*). As a secondary objective, we assessed whether functional recovery was affected by the invasiveness of open access in uncomplicated patients.

**Fig. 1:**
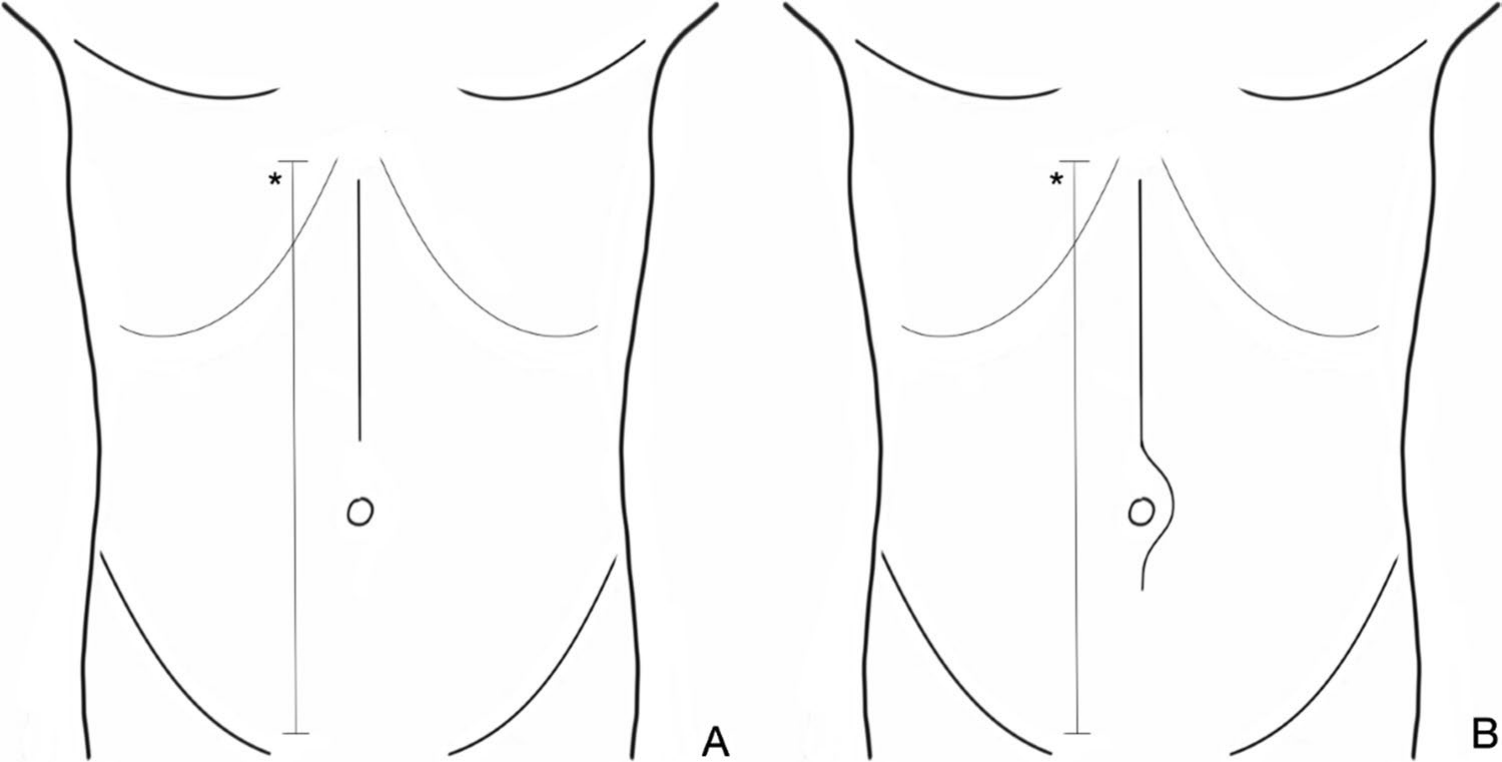
**A** Short midline incision covers < 60% of the xipho-pubic distance (*), **B** long midline incision covers ≥ 60% of xipho-pubic distance (*)

The main endpoint was time-to-functional recovery (TtFR) [13] expressed in days from index surgery to complete functional recovery. This endpoint was reached when all of the following five items were achieved: adequate pain control with oral analgesia, independent mobility, ability to maintain > 50% of the daily required caloric intake (to eat > 50% of the standard daily oral diet, provided as per the ERAS protocol), no need for intravenous fluid administration, and no signs of infection (body temperature < 38.5 °C). The invasiveness of open access was measured using the relative length of the MI to avoid biases related to sex and anthropometric parameters. According to the collected data, the average MI length was approximately 60% of the xipho-pubic distance. All MIs measuring < 60% of the xipho-pubic distance were considered less-invasive short MIs (SMIs). MIs measuring ≥ 60% of the xipho-pubic distance were considered more-invasive long MIs (LMI) (Fig. 1). SMI and LMI were defined regardless their possible extension below the umbilical region. The SMI was compared to the LMI in terms of TtFR.

Mortality and morbidity were recorded within 90 days of the index surgery and were categorized according to the Clavien-Dindo classification [14]. POPF was defined according to the updated ISGPS [15] definition as well as postpancreatectomy hemorrhage (PPH) [16] and delayed gastric emptying (DGE) [17]. The hospital stay was calculated from the day of index surgery to the day of discharge. The risk of POPF was calculated with the fistula risk score (FRS) [18]. Surgical site infection was defined according to the Centers for Disease Control and Prevention [19].

### Statistical analysis

Continuous variables are reported as the mean and standard deviation or median and interquartile range when appropriate. Differences were assessed with the Mann–Whitney test or Student’s *t* tests. Categorical variables are reported as frequencies, and differences were assessed through the chi-square test or Fisher’s exact test when appropriate. Kaplan–Meier curves were used to assess the cumulative rate of TtFR achievement. A 2-sided *p* value of less than 0.05 was considered statistically significant. Statistical analyses were carried out with SPSS software (version 20 for Mac, IBM, Chicago, IL).

## Results

From an initial population of 282 patients who underwent PD during this 1-year snapshot study, 33 were excluded because the necessary measurements were incomplete.

### Characteristics and TtFR of the study population

Perioperative characteristics and surgical outcomes of the entire study cohort (*n* = 249) are shown in Supplementary Table 1. The median age was 65 years, and the majority of patients were male. According to the relative length of the MI, we identified 138 (55.4%) patients with an SMI (median length 18 cm, range 12–24 cm) and 111 (44.6%) with an LMI (median length 23 cm, range 16–33 cm). There were no significant differences in terms of perioperative outcome between patients who underwent an LMI or an SMI, except for the fact that patients who underwent LMI were more frequently affected by arterial hypertension.

**Fig. 2:**
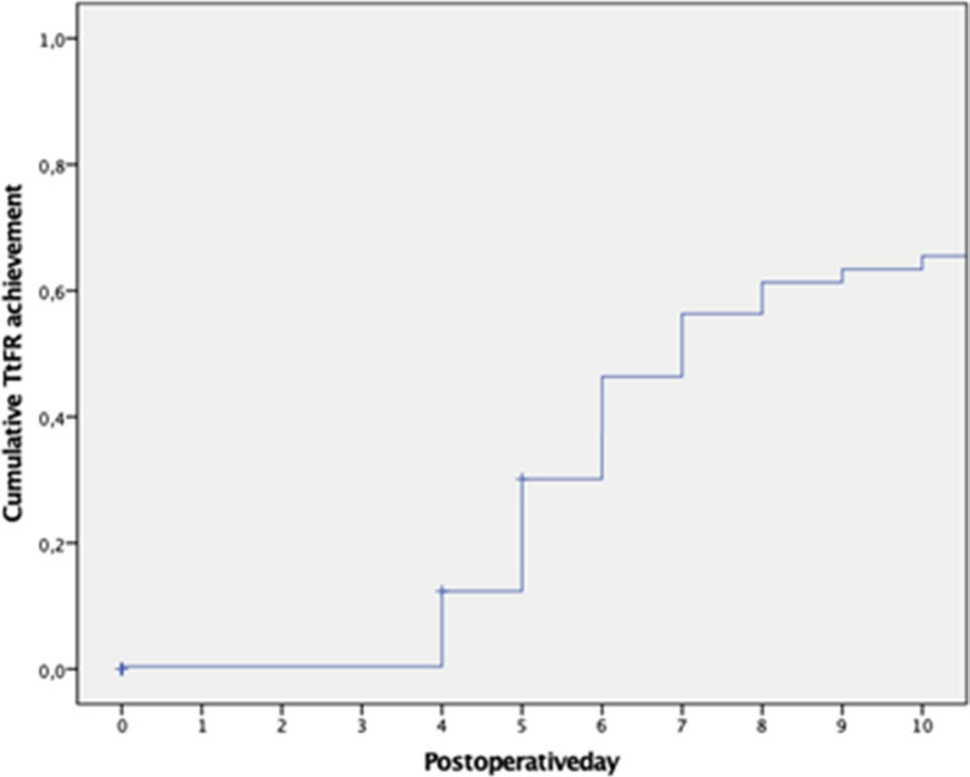
Kaplan–Meier curves showing the cumulative TtFR achievement for the entire cohort

A total of 125 (50.2%) patients developed postoperative morbidities classified as Clavien-Dindo ≥ 2. The postoperative 90-day mortality rate was 2.8%. Regardless of the development of postoperative morbidity, the overall median TtFR for open PD was 7 days, whereas the median length of hospital stay was 11 days (Table 1). Figure 2 shows Kaplan–Meier curves reporting the cumulative achievement of TtFR in the overall cohort. The overall POD6 TtFR achievement rate was 53.7%.

**Table 1:** Benchmark time to functional recovery after PD (*n* = 249)

		Overall
Length of stay (days, median, IQR)		11 (19)
Time to functional recovery (days, median, IQR)		7 (9)
Item	Pain control (days, median, IQR)	4 (2)
	Mobility (days, median, IQR)	5 (2)
	Oral food intake (days, median, IQR)	6 (7)
	Absence of signs of infection (days, median, IQR)	5 (7)

*IQR*, interquartile range

### Characteristics and TtFR of uncomplicated patients

As TtFR is affected by the development of postoperative morbidity, we excluded all patients who developed complications that required hospitalization (Clavien-Dindo ≥ 2, *n* = 125), obtaining a selected population of 124 patients (*uncomplicated patients*).

Table 2 shows the perioperative characteristics of uncomplicated patients stratified by the MI length. Patients who underwent an LMI showed a significantly higher BMI, but this was the only significant difference. Technical issues did not affect the choice between an SMI and LMI, as highlighted by the fact that there were no significant differences in terms of procedures performed after neoadjuvant therapy, vascular resections, estimated blood loss, or the time of surgery.

**Table 2:** Perioperative characteristics stratified for length of MI in uncomplicated patients (*n* = 124)

		Overall	SMI (*n* = 62)	LMI (*n* = 62)	*p*
*Preoperative*					
Age (years, median, IQR)		65 (16)	65 (13)	65 (18)	0.931
Sex	M	61 (50.8%)	29 (48.3%)	32 (53.3%)	0.715
	F	59 (49.2%)	31 (51.7%)	28 (46.7%)	
BMI (kg/m^2^, median, IQR)		23.8 (4)	23.3 (3.1)	24.5 (5.7)	0.040
Smoker		30 (25%)	20 (33.3%)	10 (16.7%)	0.057
Alcohol abuse		1 (0.8%)	1 (1.7%)	0	1.000
Diabetes		23 (19.2%)	13 (21.7%)	10 (16.7%)	0.643
Ischemic cardiac disease		2 (1.7%)	1 (1.7%)	1 (1.7%)	1.000
Hypertension		39 (32.5%)	15 (25%)	24 (40%)	0.118
COPD		0	0	0	NA
ASA score	1	5 (4.2%)	2 (3.3%)	3 (5%)	0.863
	2	96 (80%)	49 (81.7%)	47 (78.3%)	
	3	19 (15.8%)	9 (15%)	10 (16.7%)	
Neoadjuvant treatment		41 (34.2%)	19 (31.7%)	22 (36.7%)	0.701
*Intraoperative*					
Epidural analgesia		21 (17.5%)	10 (16.7%)	11 (18.3%)	1.000
Vascular resection		17 (14.3%)	6 (10%)	11 (18.6%)	0.200
Stump texture	Hard	75 (62.5%)	43 (71.7%)	32 (53.3%)	0.059
	Soft	45 (37.5%)	17 (28.3%)	28 (46.7%)	
EBL (mL, median, IQR)		530 (510)	500 (455)	550 (600)	0.203
Main duct diameter (mm, median, IQR)		4 (2)	5 (3)	4 (2)	0.196
Operative time (min, median, IQR)		420 (104)	425 (109)	420 (121)	0.769
Fistula risk zone	Negligible	5 (4.2%)	3 (5%)	2 (3.3%)	0.157
	Low	36 (30%)	21 (35%)	15 (25%)	
	Intermediate	63 (52.5%)	32 (53.3%)	31 (51.7%)	
	High	16 (13.3%)	4 (6.7%)	12 (20%)	

*ASA*, American Society of Anesthesiology; *COPD*, chronic obstructive pulmonary disease; *BMI*, body mass index; *EBL*, estimated blood loss; *IQR*, interquartile range

**Fig. 3:**
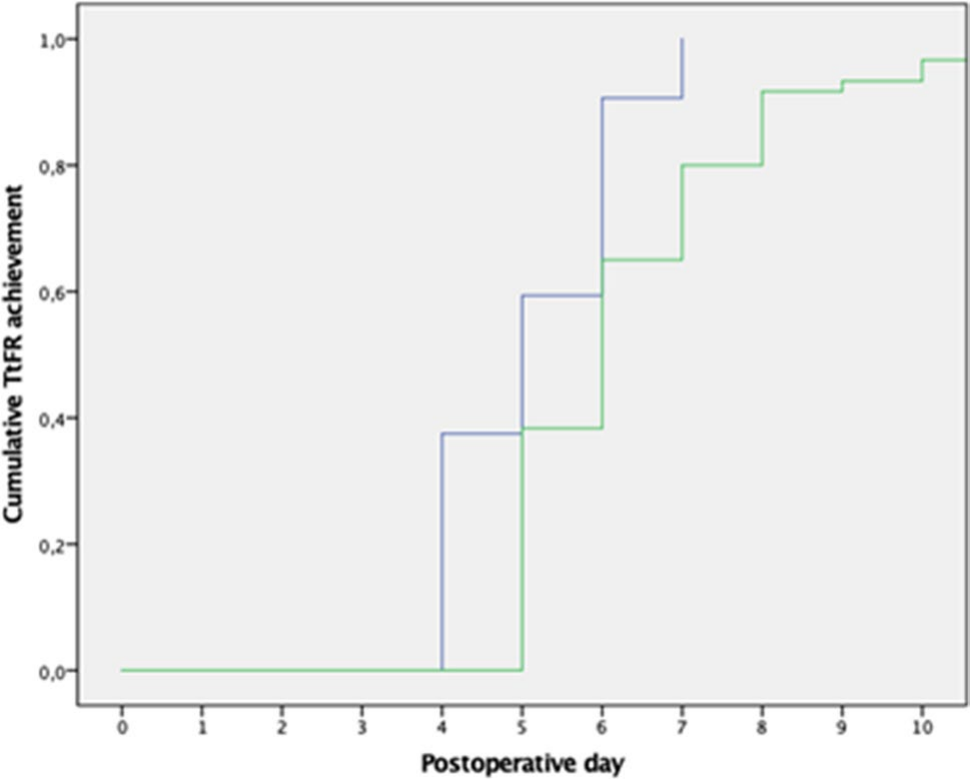
Kaplan–Meier curves showing the cumulative TtFR achievement for patients with Clavien-Dindo < 2 morbidity comparing SMI (blue) to LMI (green)

Uncomplicated patients who underwent an SMI showed significantly reduced median TtFR compared to those who underwent an LMI, particularly in terms of two items: pain control and oral food intake (Table 3). However, there was no difference in the length of hospital stay. Figure 3 shows Kaplan–Meier curves reporting the cumulative achievement of TtFR in uncomplicated patients stratified by the MI length. The POD6 TtFR achievement rate was significantly higher in patients with an SMI than with an LMI (61.7 vs. 40.6%, *p* = 0.011).

**Table 3:** Time to functional recovery stratified for length of MI in uncomplicated patients (*n* = 124)

		Overall	SMI (*n* = 62)	LMI (*n* = 62)	*p*
Length of stay (days, median, IQR)		7 (3)	8 (3)	7 (3)	0.775
Time to functional recovery (days, median, IQR)		6 (2)	5 (2)	6 (2)	0.002
Items	Pain control (days, median, IQR)	4 (1)	4 (1)	5 (1)	0.048
	Mobility (days, median, IQR)	4 (1)	4 (1)	5 (1)	0.114
	Oral food intake (days, median, IQR)	5 (1)	5 (2)	6 (1)	0.001
	Absence of signs of infection (days, median, IQR)	4 (1)	4 (1)	4 (1)	0.830

*IQR*, interquartile range

## Discussion

The present study depicted the time to functional recovery after open PD with an MI. Regardless of postoperative morbidity, half of the patients achieved satisfactory recovery within 1 week from surgery, which can be considered as a benchmark independently of the actual length of hospital stay. Moreover, among uncomplicated patients, functional recovery seemed to be affected by the magnitude of access to the abdominal cavity, as patients who received a small incision recovered earlier.

The length of hospital stay has always been considered the main indicator of functional recovery, and it is considered among the key outcome metrics after PD [1, 2]. Compared to low-volume hospitals, centers of excellence report a median hospital stay of 11 days, and most of their postoperative clinical pathways are targeted to achieve discharge from the hospital within 1 week of surgery [20]. We hereby reported a similar median length of hospital stay, yet the relevance of this parameter is questioned because it depends on multiple factors. These include the healthcare organization, the availability of rehabilitation facilities, and cultural aspects that do not allow a fair comparison between different realities worldwide. Indeed, despite showing comparable postoperative mortality rates, a large retrospective series from different countries reported relevant differences in terms of median hospital stay [21–23]. For these reasons, TtFR appears to be a more objective parameter than hospital stay suitable for measuring and comparing the outcomes of centers performing pancreatic surgery.

Most surgeons worldwide still perform PD with “classic” open access, either via midline or transverse incisions. However, minimally invasive PD has gained great popularity, developed rapidly, and produced intriguing but variable results. Numerous single-institution studies, systematic reviews, and meta-analyses have been published, revealing remarkable selection biases [5, 6]. These studies have shown promising results in terms of reduced intraoperative bleeding and faster postoperative recovery sufficient to suggest that the high costs of minimally invasive PD could be offset by reductions in postoperative hospitalization costs. Few randomized trials have been published and have shown inconsistent results in terms of both surgical and oncological outcomes. Palanivelu et al. [24] randomized 64 patients to open or minimally invasive PD, showing a significantly lower amount of intraoperative blood loss and length of hospital stay for the minimally invasive approach. However, the open PD group had a median length of stay of 13 days, which was quite higher than that usually reported by high-volume centers, as in the present study. The PADULAP trial [25], on the other hand, randomized 66 patients and reported that minimally invasive PD was associated with a significantly reduced comprehensive complication index, reduced poor quality outcome, and a reduced rate of severe morbidity. Even in this trial, the authors reported a reduced hospital stay for the minimally invasive approach, but the benchmark was a group of open PDs with a median hospital stay of 17 days. The LEOPARD-2 trial [13] included 99 patients and reported interesting data using TtFR. Even though the trial was prematurely terminated due to safety reasons, the authors found a nonsignificant difference between open and minimally invasive PD in terms of TtFR, but the latter group demonstrated a median TtFR of 10 days. Of note, this is 3 days longer than the length in the present series.

These data attest that a reduction in postoperative hospitalization and related costs after elective PD with the minimally invasive approach is not supported by robust evidence. In centers with great familiarity with advanced laparoscopy, the lack of experience and standardization of clinical pathways after major pancreatic resection could result in delayed recovery and prolonged hospitalization even after minimally invasive PD. On the other hand, in centers of excellence for pancreatic surgery with solid multidisciplinary support and consolidated experience in postoperative management, minimally invasive and open PD could have completely overlapping short-term postoperative outcomes.

Further studies are therefore needed to better clarify the possible advantages of minimally invasive PD. However, it is preliminarily necessary to choose the most suitable outcome metrics for postoperative recovery, taking into account all the concerns highlighted for the length of hospital stay. Moreover, we must reflect on the appropriateness of the groups being compared, as we need to define a standardized benchmark for open PD that could be the one performed in a high-volume center with a small open access.

The current study has several limitations. This was a single-center prospective snapshot experience, and our institutional protocols for postoperative management could have influenced TtFR, affecting the reproducibility of the results in different settings. Unmeasurable factors could have influenced TtFR, such as the presence of patients with a more proactive attitude than others, the availability of nursing and rehabilitation staff to encourage early patient mobilization, or different pain thresholds that could have affected the discontinuation of intravenous analgesia. Certainly, the length of hospital stay has been influenced by well-known issues, as discharge planning depends on patient turnover in the surgical ward, patient country/region of origin, and behavioral and cultural factors. Further unmeasurable factors could have affected the choice between an SMI and LMI. Even if there were no differences among most important predictors of a complicated postoperative course, the choice of an LMI could have selected patients with delayed recovery due to more challenging procedures requiring a wider surgical field. Moreover, despite not statistically significant, there were some differences between the cohorts which could have influenced the results (e.g., more vascular resections, more soft glands, and a higher FRS in the LMI group). Finally, however unlikely, the non-significant differences in terms of PPH and mortality rates found in the present study may be of concern with regard to the safety of SMI, if confirmed by future studies with appropriate calculation of sample size.

## Conclusions

In this snapshot prospective study, half of the patients undergoing open elective PD at a high-volume center achieved satisfactory functional recovery within 1 week of surgery. This result was reproduced regardless of the occurrence of complications, and this approach should be considered the correct benchmark in future studies comparing minimally invasive with open PD. In uncomplicated patients, functional recovery seems to be affected by the magnitude of the open approach, as individuals with an SMI recover faster than those with an LMI.

## Supplementary Information

Below is the link to the electronic supplementary material.

Supplementary file1 (DOCX 19 KB)
