# Supplementary material for: Open pancreaticoduodenectomy: setting the benchmark of time to functional recovery
Source: Langenbecks Arch Surg. 2021 Sep 23;407(3):1083–9. doi: 10.1007/s00423-021-02333-3 (PMC9151571; doi:10.1007/s00423-021-02333-3)
Supplement: Supplementary file 1 — Supplementary file1 (DOCX 19 KB) [file 423_2021_2333_MOESM1_ESM.docx]

| **Supplementary Table 1 – Perioperative characteristics and surgical ouctome of the entire population (n= 249)** | | | | | |
| --- | --- | --- | --- | --- | --- |
|  | | **Overall** | **SMI**  **(n= 138)** | **LMI**  **(n= 111)** | **p** |
| **Age (years, median, IQR)** | | 65 (14) | 65 (14) | 65 (16) | 0.936 |
| **Sex** | **M** | 133 (53.4%) | 75 (54.3%) | 58 (52.3%) | 0.799 |
|  | **F** | 116 (46.6%) | 63 (45.7%) | 53 (47.7%) |  |
| **BMI (Kg/m^2^, median, IQR)** | | 24.4 (5) | 23.8 (4) | 25.3 (6) | 0.118 |
| **Smoker** | | 61 (24.5%) | 40 (29%) | 21 (18.9%) | 0.076 |
| **Alcohol abuse** | | 5 (2%) | 5 (3.6%) | 0 | 0.067 |
| **Diabetes** | | 42 (16.9%) | 25 (18.1%) | 17 (15.3%) | 0.612 |
| **Comorbidities** | | 151 (60.6%) | 83 (60.1%) | 68 (61.3%) | 0.897 |
| **Ischemic cardiac disease** | | 8 (3.2%) | 6 (4.3%) | 2 (1.8%) | 0.305 |
| **Hypertension** | | 93 (37.3%) | 42 (30.4%) | 51 (45.9%) | 0.013 |
| **COPD** | | 2 (0.8%) | 2 (1.4%) | 0 | 0.504 |
| **ASA score** | **1** | 6 (2.4%) | 3 (2.2%) | 3 (2.7%) | 0.955 |
|  | **2** | 190 (76.3%) | 106 (76.8%) | 84 (75.7%) |  |
|  | **3** | 53 (21.3%) | 29 (21%) | 24 (21.6%) |  |
| **Neoadjuvant treatment** | | 80 (32.1%) | 44 (31.9%) | 36 (32.4%) | 1.000 |
| **Vascular resection** | | 35 (14.1%) | 18 (13%) | 17 (15.6%) | 0.586 |
| **Stump texture** | **Hard** | 113 (45.4%) | 68 (49.3%) | 45 (40.5%) | 0.201 |
|  | **Soft** | 136 (54.6%) | 70 (50.7%) | 66 (59.5%) |  |
| **EBL (mL, median, IQR)** | | 592 (500) | 530 (450) | 600 (570) | 0.069 |
| **Main duct diameter (mm, median, IQR)** | | 4 (3) | 4 (3) | 4 (2) | 0.730 |
| **Operative time (min, median, IQR)** | | 430 (121) | 430 (113) | 440 (125) | 0.253 |
| **Fistula risk zone** | **Negligible** | 8 (3.2%) | 6 (4.4%) | 2 (1.8%) | 0.422 |
|  | **Low** | 53 (21.3%) | 33 (24.1%) | 20 (18.2%) |  |
|  | **Intermediate** | 131 (52.6%) | 69 (50.4%) | 62 (56.4%) |  |
|  | **High** | 55 (22.1%) | 29 (21.2%) | 26 (23.6%) |  |
| **POPF** | | 55 (22.1%) | 30 (21.9%) | 25 (22.5%) | 1.000 |
|  | **B** | 50 (20.1%) | 27 (19.6%) | 23 (20.7%) | 0.776 |
|  | **C** | 5 (2%) | 3 (2.2%) | 2 (1.8%) |  |
| **Abscess** | | 43 (17.3%) | 27 (19.6%) | 16 (14.4%) | 0.315 |
| **Biliary fistula** | | 17 (6.8%) | 9 (6.5%) | 8 (7.2%) | 1.000 |
| **PPH** | | 48 (19.3%) | 32 (23.5%) | 16 (14.4%) | 0.077 |
|  | **A** | 10 (4%) | 5 (3.6%) | 5 (4.5%) | 0.179 |
|  | **B** | 32 (12.9%) | 22 (15.9%) | 10 (9%) |  |
|  | **C** | 6 (2.4%) | 5 (3.6%) | 1 (0.9%) |  |
| **DGE** | | 66 (26.5%) | 37 (27.4%) | 29 (26.6%) | 1.000 |
|  | **A** | 15 (6%) | 9 (6.5%) | 6 (5.4%) | 0.625 |
|  | **B** | 40 (15.7%) | 20 (14.5%) | 19 (17.1%) |  |
|  | **C** | 11 (4.4%) | 8 (5.8%) | 3 (2.7%) |  |
| **Sepsis** | | 49 (19.7%) | 31 (22.5%) | 18 (16.2%) | 0.262 |
| **Post-operative pneumonia** | | 45 (18.1%) | 24 (17.4%) | 21 (18.9%) | 0.869 |
| **Urinary tract infection** | | 8 (3.2%) | 6 (4.3%) | 2 (1.8%) | 0.305 |
| **Acute kidney injury** | | 14 (5.6%) | 8 (5.8%) | 6 (5.4%) | 1.000 |
| **Surgical site infection** | | 34 (13.7%) | 17 (12.3%) | 17 (15.3%) | 0.578 |
| **Post-operative transfusion** | | 54 (21.7%) | 32 (23.4%) | 22 (19.8%) | 0.539 |
| **Relaparotomy** | | 24 (9.6%) | 18 (13%) | 6 (5.5%) | 0.052 |
| **ICU not planned** | | 30 (12%) | 19 (13.8%) | 11 (9.9%) | 0.435 |
| **Readmission** | | 9 (3.6%) | 6 (4.3%) | 3 (2.7%) | 0.735 |
| **Clavien-Dindo** | **I** | 83 (33.3%) | 50 (36.2%) | 33 (29.7%) | 0.095 |
|  | **II** | 73 (29.3%) | 41 (29.7%) | 32 (28.8%) |  |
|  | **IIIA** | 17 (6.8%) | 9 (6.5%) | 8 (7.2%) |  |
|  | **IIIB** | 10 (4%) | 7 (5.1%) | 3 (2.7%) |  |
|  | **IVA** | 12 (4.8%) | 7 (5.1%) | 5 (4.5%) |  |
|  | **IVB** | 6 (2.4%) | 4 (2.9%) | 2 (1.8%) |  |
|  | **V (mortality)** | 7 (2.8%) | 6 (4.3%) | 1 (0.9%) |  |

ASA: American Society of Anesthesiology; COPD: chronic obstructive pulmonary disease; BMI: body mass index; DGE: delayed gastric emptying; EBL: estimated blood loss; ICU: intensive care unit; IQR: interquartile range; POPF: postoperative pancreatic fistula; PPH: postpancreatectomy hemorrhage.
